# Photon antibunching control in a quantum dot and metallic nanoparticle hybrid system with non-Markovian dynamics

**DOI:** 10.1038/s41598-018-29799-4

**Published:** 2018-08-20

**Authors:** T. Moradi, M. Bagheri Harouni, M. H. Naderi

**Affiliations:** 1 Department of Physics, Faculty of Science, University of Isfahan, Hezar Jerib Str., Isfahan, 81746-73441 Iran; 2 Quantum Optics Group, Department of Physics, Faculty of Science, University of Isfahan, Hezar Jerib Str., Isfahan, 81746-73441 Iran

## Abstract

Photon-number statistics of the emitted photons from a quantum dot placed in the vicinity of a metallic nanoparticle driven by a laser in the non-Markovian regime is investigated theoretically. In the model scheme, the quantum dot is considered as an InAs three-level system in L-type configuration with two transition channels. We aim to introduce the hybrid system as a nonclassical photon source and control the antibunching behavior of the emitted photons by the geometrical as well as the physical parameters of the hybrid system. Our approach is based on the classical Green’s function technique and time convolution master equation. The results reveal that the emitted photons from the hybrid system under consideration are antibunched and energy is exchanged between the QD and nanoshell. By increasing the QD-MNP separation distance, the detuning frequency between the QD transitions and surface plasmon modes, and the Rabi frequency the antibunching time increases while the backaction of the reservoir on the QD decreases. To sum up, we conclude that the studied system has the potential to be a highly controllable single-photon source.

## Introduction

Nonclassical states of light possess important role in quantum optics and quantum technologies. Single-photon sources, as one of most prominent types of nonclassical emitters and as the crucial ingredient of quantum information technology are amongst the most widely investigated quantum systems during the last four decades or so^1–4^. Numerous applications for single-photon sources have been proposed in the fields of quantum cryptography^5,6^, quantum repeaters^7,8^, and quantum computation^9^. In addition, the low-noise nature of nonclassical photons makes them as ideal candidates for application in the fundamental measurement problems^2^. To date, various theoretical schemes and experimental demonstrations have been carried out on the generation of nonclassical states of light. Some examples include atomic cascade transition in calcium atoms^10^, single ions in traps^11^, parametric down-conversion^12,13^, single molecules coupled to a resonant cavity^14^, semiconductor quantum dots (QDs)^4,15–17^, defect centers in diamond^18^, single-walled carbon nanotubes^19^, and photonic crystals^20^.

The nonclassical-light or single-photon sources are not limited to the above mentioned systems and setups. Another way of realizing an on-demand single photon source is to use the hybrid systems composed of a semiconductor QD and a metal nanoparticle (MNP)^21,22^. The presence of the MNP near an emitter changes the environment density of states around the emitter. It is well known that the environment of an emitter influences its decay rate (Purcell effect)^23^. The interaction of an MNP^24^ or graphene^25^ with light leads to non-propagating excitations of the conduction electrons of the MNP; the quanta of these excitations are called localized surface plasmons (LSPs). The evanescent near-field associated^26^ to the LSPs increases the local density of states (LDOS) around the MNP dramatically^22,27–32^. When a QD, as an emitter, is located in the evanescent field of an MNP, the decay rate of the QD is affected through the LDOS and increases significantly^27–29,32^. The physical features of the QD-MNP system can be controlled by the geometry of the hybrid system, i.e., the shape of the MNP and the QD-MNP separation distance. Depending on the geometry, the system can operate either in the strong-coupling or the weak-coupling regime and exhibits the non-Markovian^27,33^ or Markovian behavior^30,34^.

In recent years, significant theoretical studies have been performed on the photon-number statistics of emitted light from a variety of hybrid systems in order to provide efficient methods for the controllable generation of antibunched photons. In ref.^35^ the conditions for the realization of photon antibunching of molecular fluorescence in a hybrid system of a single molecule and a plasmonic nanostructure composed of four nanostrips have been theoretically investigated by using the Green’s tensor technique^36^. The photon-number statistics from the resonance fluorescence of a two-level atom near a metallic nanosphere driven by a laser field with finite bandwidth has also been studied, and it has been shown that the statistics can be controlled by the location of the atom around the metal nanosphere, the intensity and the bandwidth of the driving laser, and detuning from the atomic resonance^37^. In addition, a theoretical framework based on the combination of the field-susceptibility/Green’s-tensor technique with the optical Bloch equations has been developed^38^ to describe the photon statistics of a quantum system coupled with a complex dielectric or metallic nanostructures. By using an approach based on the Green’s function method and a time-convolutionless master equation, the dynamics of the photon-photon correlation function in a hybrid system composed of a solid-state qubit placed near an infinite planar surface of a dissipative metal has been studied^21^ under the Markov approximation. For a hybrid structure consisting of an optically driven two-level QD coupled to a metallic nanoparticle cluster it has been shown^39^ that the single-photon emission can be efficiently controlled by the geometrical parameters of the system.

In this paper, we theoretically investigate the photon-number statistics of the light emitted from a single semiconductor QD with Λ-type configuration in the vicinity of a metal nanoshell in the non-Markovian regime. The present study follows two main purposes. First, we aim to introduce the hybrid system as a nonclassical photon source. Second, we intend to investigate whether the photon-number statistics can be controlled by the geometrical and physical parameters of the hybrid system. Our theoretical description of the system involves the quantization of electromagnetic field in the presence of an MNP within the framework of the classical dyadic Green’s function approach including quantum noise sources which is appropriate for dispersive and absorbing media.

The ohmic nature of metals has a substantial impact on the reduction of the effective QD-MNP interaction, specially at high frequencies due to the interband transitions. One effective way to deal with this phenomenon is to use geometries in which the surface plasmons are induced in low energies. The plasmon resonance frequencies of a nanoshell are adjustable by the thickness of the nanoshell and the dielectric permittivities of the core and the shell materials. Therefore, the resonance frequency modes of a nanoshell can appear at much lower energies than those of a nanosphere^40–46^. Therefore, in a nanoshell sphere geometry the interband transitions have small impacts in comparison with a sphere geometry. Thus in the present contribution we study a hybrid system consisting of a QD in the proximity of a metal nanoshell.

The paper is structured as follows. Numerical results and discussions are presented in first, where we explore the controllability of the photon-number statistics through the physical as well as the geometrical parameters of the hybrid system. In the next part, we present our conclusions. Finally, we describe the theoretical model of the hybrid system composed of a single QD coupled to an MNP within the framework of the master equation approach. Then, we derive an expression for the normalized second-order autocorrelation function for the photons emitted by the QD.

## Results and Discussion

**Figure 1:**
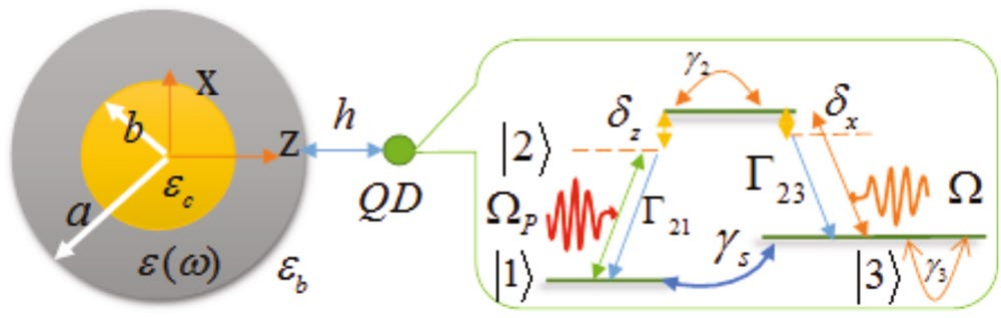
Schematic illustration of the QD-MNP hybrid system under consideration. A three-level Λ-type configuration QD, with z- and x-oriented dipole moments, is located at distance *h* away from the outer surface of a silver nanoshell composed of a spherical core of radius *a* and permittivity *ε*_*c*_ surrounded by a concentric Ag shell of radius *b* and frequency-dependent permittivity *ε*(*ω*), embedded in a medium with permittivity *ε*_*b*_. The transition $\mathrm{|2}\rangle \leftrightarrow \mathrm{|3}\rangle$ is coupled to the *x*-polarized driving field with Rabi frequency Ω, and detuning Δ. On the other hand, it couples to the x component of surface plasmon modes on the MNP with detuning *δ*_*x*_. The transition $\mathrm{|2}\rangle \leftrightarrow \mathrm{|1}\rangle$ is coupled to the elementary *z*-excitations of the MNP with frequency Ω_*p*_ and detuning *δ*_*z*_.

As shown in Fig. 1, the physical system consists of a QD which is located at distance *h* from the surface of a nanoshell of inner radius *b*, outer radius *a*, and frequency-dependent permittivity *ε*(*ω*). The QD-nanoshell system is embedded in a homogeneous background medium with relative permittivity *ε*_*b*_. As a realistic example similar to refs^47–49^, we choose a Λ-type three-level InAs QD with permittivity *ε*_*QD*_ = 11.58^50,51^. The discrete energy states $\mathrm{|2}\rangle$ and $\mathrm{|3}\rangle$ have energies $\hslash {\omega }_{2}$ and $\hslash {\omega }_{3}$, respectively, with respect to the state $\mathrm{|1}\rangle$, such that $\hslash {\omega }_{2} > \hslash {\omega }_{3} > \hslash {\omega }_{1}$.

It has been shown experimentally^51^ that the dipole moments *d*_21_ and *d*_23_ in the InAs QD are aligned perpendicular to each other^52^. Thus, the atomic dipole moment operator is taken as $d=d(\mathrm{|2}\rangle \langle \mathrm{1|}z+\mathrm{|2}\rangle \langle \mathrm{3|}x)+H\mathrm{.}c.$ in which *d* is real. Moreover, we consider the classical driving field as a *x*-polarized field. Therefore, the transition $\mathrm{|2}\rangle \leftrightarrow \mathrm{|3}\rangle$ is coupled to the classical driving field with Rabi frequency Ω, and detuning Δ. On the other hand, this transition also couples to the *x* component of surface plasmon modes on the MNP with detuning *δ*_*x*_. The transition $\mathrm{|2}\rangle \leftrightarrow \mathrm{|1}\rangle$ is coupled to the elementary *z*-excitations of the MNP with frequency Ω_*p*_ and detuning *δ*_*z*_. The classical driving field does not couple to the transition $\mathrm{|2}\rangle \leftrightarrow \mathrm{|1}\rangle$ because they are aligned along the perpendicular directions. In this section, we present and discuss various numerical results and calculations to analyze the quantum statistics of the photons emitted from the hybrid QD-MNP system under consideration (Fig. 1). Throughout the calculations, we use atomic units ($\hslash =1$, 4*πε*_0_ = 1, *c* = 137, and *e* = 1). For the numerical calculations, we consider a three-level self-assembled InAs QD as the emitter with *z*- and *x*-oriented dipole moments of |*d*_21_| = |*d*_23_| = 0.1 *e* nm and *ω*_21_ = 0.8046 eV and *ω*_23_ = 0.8036 eV^51^. The QD is placed at distance *h* from the outer surface of a silver nanoshell composed of a spherical core of radius *a* and permittivity *ε*_*c*_ surrounded by a concentric Ag shell of radius *b* and frequency-dependent permittivity *ε*(*ω*), embedded in a medium with permittivity *ε*_*b*_.

An important ingredient for our numerical calculation is the dielectric permittivity of MNP. At low photon frequencies below the interband transitions region, the permittivity function of metals can be well described by the Drude model. For silver the interband effects already start to occur for energies in excess of 1 eV and thus the validity of the Drude model breaks down at high frequencies^53,54^. Therefore, in the numerical calculations we will use the measured dielectric data of silver reported in ref.^54^ in which the plasma frequency of the bulk *ω*_*p*_ = 9.2 *eV* and the collision rate of the free electrons *γ*_*bulk*_ = 0.021 *eV*^55^. Moreover, we have included the size effect which increases the damping rate of the electrons as *γ* = *γ*_*bulk*_ + *Av*_*F*_/*l*. Here, *v*_*F*_ is the Fermi velocity (approximately 1.4 × 10^6^ *m*/*s* in silver) and *l* depends on the geometry of the nanoparticle, which in a nanoshell is the thickness of the shell. The parameter *A* is determined by the geometry and theory, which for silver has the value *A* = 0.25^56^. The frequency- and size-dependent dielectric function of a noble metal by taking into account the size effect would be expressed as^57^

1 $$\varepsilon (\omega ,l)={\varepsilon }_{bulk}+\frac{{\omega }_{p}^{2}}{{\omega }^{2}+i\omega {\gamma }_{bulk}}-\frac{{\omega }_{p}^{2}}{{\omega }^{2}+i\omega \gamma }.$$

### LDOS of the system

Density of states which changes locally around the MNP provides us good insights about the interaction of the QD-MNP and the frequencies of surface plasmons which depend strictly on the orientation of the dipole moment of the QD. The scaled LDOS of a hybrid system is defined as ${\rho }_{zz(xx)}/{\rho }_{0}=$ ${\mathbb{I}}{\rm{m}}[{G}_{zz(xx)}({r}_{d},{r}_{d},\omega )]/{\rho }_{0}$ in which *ρ*_0_ = *k*_1_/6*π* being the free space density of states^31,58^. In this relation *G*_*zz*(*xx*)_ is the *zz*(*xx*)-component of the dyadic Green’s function of the system.

**Figure 2:**
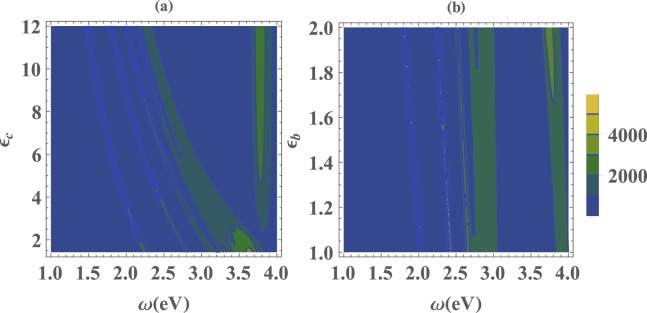
Density plot of the scaled LDOS of (**a**) a (20,16)nm nanoshell versus frequency *ω* and the dielectric permittivity of the core *ε*_*c*_ when *ε*_*b*_ = 1.78 and (**b**) a (20, 16) nm nanoshell versus frequency *ω* and the dielectric permittivity of the background medium *ε*_*b*_ when *ε*_*c*_ = 5.4.

In Fig. 2 we have plotted the scaled LDOS, *ρ*_*zz*_/*ρ*_0_, to examine the influence of the core and medium materials on the resonance frequencies of the plasmon modes. As can be seen in Fig. 2(a), with increasing the core dielectric permittivity (*ε*_*c*_) the resonance frequencies of plasmon modes shift toward lower frequencies. Similarly, Fig. 2(b) shows that the resonance frequencies of the plasmon modes experience a slight redshift once we fix the core dielectric (*ε*_*c*_ = 5.4) and change the embedding medium dielectric constant. These results are in agreement with the experimental findings reported in ref.^42^.

In general, the results show that increasing the dielectric permittivities of the core and the embedding medium lead to the redshift of the plasmon resonances. This behavior has a simple physical interpretation. As is well known, within the quasi-static approach, surface plasmons are collective electromagnetic oscillations at metallic surfaces over a fixed positively charged background, with induced surface charges providing the restoring force. Increasing the dielectric permittivity of the surrounding medium causes the strength of the surface charges to be effectively reduced, leading to a decreased restoring force and consequently, the plasmon energies are lowered. By increasing the core and the medium relative permittivities we adjust the surface plasmon resonance on demand frequency. By tuning the dielectric functions of background and core of the nanoshell to *ε*_*b*_ = *ε*_*c*_ = 10.95^50^ the surface plasmons resonance frequencies for *ω*_*n*−_ with *n* = 1 is 0.8026 *eV* for both *z* and *x* surface plasmons which can couple effectively with InAs QD^51^.

**Figure 3:**
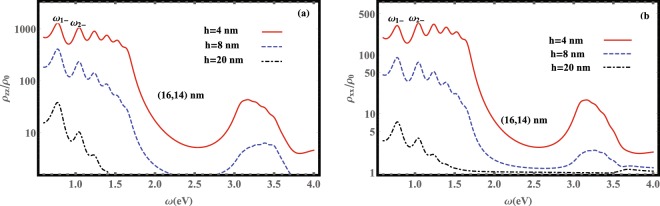
Scaled LDOS, (**a**) *ρ*_*zz*_/*ρ*_0_, (**b**) *ρ*_*xx*_/*ρ*_0_, versus frequency *ω* for a (16, 14) nm nanoshell for different values of *h*.

In Fig. 3(a) and (b) we have plotted the scaled LDOS for different QD-MNP separation distances of a (16, 14) nm nanoshell composed of a spherical *GaAs* core of radius *b* = 14 nm and permittivity *ε*_*c*_ = 10.95^59^ surrounded by a concentric Ag shell of radius *a* = 16 nm and frequency-dependent permittivity *ε*(*ω*), embedded in a medium with permittivity *ε*_*b*_ = 10.95^59^.

The resonance frequencies of surface plasmons can be obtained by the poles of the dyadic Green’s function. The features of the plasmon resonances in a metallic nanoshell are described by the plasmon hybridization model^60^. In this model, the surface plasmon modes of internal and external surfaces (or core and shell surfaces) are combined in two symmetric and antisymmetric modes. Accordingly, these new branch modes possess low (${\omega }_{n-}^{z(x)}$) and high (${\omega }_{n+}^{z(x)}$) energies. The thickness of the nanoshell controls the strength of the interaction between the sphere-like and the cavity-like plasmon modes. With increasing the thickness of the nanoshell these modes behave more independently. The important parameters, which possess important influences on the plasmon resonance frequency, are the thickness of MNP (*a* − *b*), the core material dielectric constant (*ε*_*c*_), and the embedding medium dielectric constant (*ε*_*b*_).

According to the hybridization model the plasmon peaks are grouped into two basic branches, i.e., ${\rm{\Sigma }}{\omega }_{n-}$ (plasmon peaks on the left side of Fig. 3) and ${\rm{\Sigma }}{\omega }_{n+}$ (plasmon peaks on the right side of Fig. 3). The first left peak belongs to ${\omega }_{n-}$ with *n* = 1^40^. As can be seen from Fig. 3(a) and (b) for a fixed aspect ratio *b*/*a* the resonance frequencies of plasmon modes are scale-invariant, i.e., although the intensities of plasmon modes get decreased by increasing the QD-MNP separation distance *h*, the energy arrangement remains unchanged for different values of *h*. Thus, by controlling the aspect ratio *b*/*a* as well as the materials of the core and medium, one can tune the plasmon resonance frequency on demand.

Figure 3(a) shows the enhancement of LDOS due to coupling of the *z*-oriented dipole moment of the QD, i.e., the transition $\mathrm{|2}\rangle \leftrightarrow \mathrm{|1}\rangle$, to the MNP. Similarly, Fig. 3(b) indicates the coupling of the *x*-oriented dipole moment of the QD, i.e., the transition $\mathrm{|2}\rangle \leftrightarrow \mathrm{|3}\rangle$, to the MNP. Making a comparison between Fig. 3(a,b) reveals that the QD-MNP coupling depends on the orientation of the transition dipole moment of the QD. In the other words, the MNP affects differently on electromagnetic modes with perpendicular polarizations. Thus, the enhancement of LDOS is different for *x*- and *z*-directions and anisotropic Purcell effect happens^32^.

### Spontaneous decay of the QD excited state

**Figure 4:**
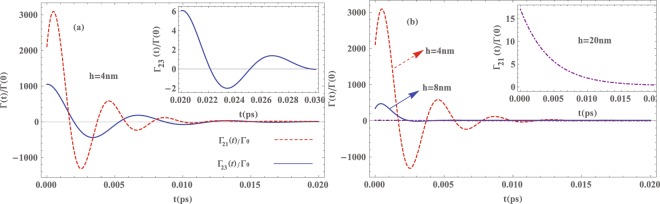
Time evolution of the spontaneous decay rate of a QD placed in the vicinity of a (16, 14) nm nanoshell. (**a**) Γ_21_(*t*), Γ_23_(*t*) when the QD is placed at a distance 4 nm from the MNP. (**b**) Γ_21_(*t*) for different values of the QD-MNP separation distance (*h*). The inset in (**a**) represents the long-time relaxation behavior of Γ_23_(*t*)/Γ_0_ and in (**b**) shows Γ_21_(*t*)/Γ_0_ for *h* = 20 nm. Here, the permittivities of the core and the embedding medium are assumed to be equal, *ε*_*c*_ = *ε*_*b*_ = 10.95.

It is well known that one of the obvious evidences for the non-Markovian character of relaxation processes such as spontaneous decay of an excited state of a given quantum system is the nonexponential time evolution due to the memory effects. Here, we numerically examine the memory effects on the spontaneous decay of the QD in the system under consideration. For this purpose, we assume that the QD is initially prepared in the excited state $\mathrm{|2}\rangle$ and we set Ω = 0, i.e., there is an exciton in the QD and no plasmon mode in the MNP. In Fig. 4 we have plotted the scaled spontaneous decay rates of the excited state $\mathrm{|2}\rangle$ for both transitions $\mathrm{|2}\rangle \to \mathrm{|1}\rangle$ and $\mathrm{|2}\rangle \to \mathrm{|3}\rangle$, i.e., Γ_21_(*t*)/Γ_0_ and Γ_23_(*t*))/Γ_0_ (determined by the real parts of the inverse Laplace transforms of the parameters *β*_*z*_(*s*), ${\eta }_{x}^{\mathrm{(1)}}(s)$, ${\eta }_{x}^{\mathrm{(2)}}(s)$ and *α*_*x*_(*s*) given in Eq. (14)), versus time for different separation distances between the QD and a (16, 14) nm nanoshell. Here, ${{\rm{\Gamma }}}_{0}=({d}^{2}{\omega }_{2}^{3})/(3\pi {\varepsilon }_{0}\hslash {c}^{3})$ refers to the free-space decay rate of the QD whose typical value is about 10^6^ *s*^−1^ ^24^. We assume that the detuning between the QD transition $\mathrm{|2}\rangle \to \mathrm{|1}\rangle$ and the surface plasmon mode is near zero ($\delta \simeq 0$) that is the emitter is near resonance with the localized surface plasmon modes and ${\omega }_{1-}^{z}$ = 0.8026 eV (see Fig. 3(a)). The excited QD couples to the MNP and excites a plasmon mode which is nearly on resonance with its energy. The excited resonance plasmon mode re-excites the QD before dissipation to other modes. The oscillatory behavior of Γ(*t*) indicates the re-excitation of the QD after a finite delay time by the reservoir which is the signature of non-Markovian dynamics of the system under study. As can be seen from Fig. 4(a), Γ_21_ which corresponds to the decay rate of transition $\mathrm{|2}\rangle \to \mathrm{|1}\rangle$ shows completely the non-Markovian dynamics of this channel which is directed along the *z* axis. Moreover, small oscillations of spontaneous decay rate of $\mathrm{|2}\rangle \to \mathrm{|3}\rangle$ which is directed along the *x* axis indicates moderate non-Markovian dynamics of this channel. In Fig. 4(b) we have plotted the spontaneous decay rate of transition $\mathrm{|2}\rangle \to \mathrm{|1}\rangle$ for *h* = 4, 8, and 20 nm. This figure shows that with increasing the QD-MNP separation distance the occupation probability of the QD excited state decays slower in time and its dynamics tends to the Markovian regime for large enough QD-MNP distance. These results are fully consistent with those obtained from Fig. 4(a). As can be seen from Fig. 4(a) and (b), the QD spontaneous decay rates exhibit a damped oscillatory behavior in the course of time evolution and eventually tend to the Markovian decay rate Γ_0_ in both channels. It is worth to note that the negative values of decay rates indicate the slow down of the spontaneous decay of the excited state. Physically, this behavior occurs as the result of the reservoir backaction on the QD which reflects the non-Markovian nature of the dynamics. Furthermore, with increasing the QD-MNP separation distance the amplitude of oscillations decreases such that for *h* = 20 nm, the decay rate Γ(*t*) shows no oscillations (the inset plot of Fig. 4(b)). This is because with increasing the QD-MNP distance, the intensity of the LDOS decreases and consequently, the emitter QD experiences a less structured reservoir.

### Single-photon emission by the three-level QD

Here, we are going to investigate the photon-number statistics to better understand the features of the emitted light by a single emitter (QD) in the vicinity of an MNP. The photon-number statistics as well as the quality of a single-photon source are determined by measuring the normalized second-order autocorrelation function of photons, *g*^(2)^(*τ*), in a Hanbury Brown-Twiss (HBT) setup^61^. The general criterion for photon antibunching is ${g}^{\mathrm{(2)}}(\tau ) > {g}^{\mathrm{(2)}}\mathrm{(0)}$. Although in the ideal case *g*^(2)^(0) = 0 indicates the antibunching of photons, a value of ${g}^{\mathrm{(2)}}\mathrm{(0)} < 0.5$ is generally accepted as a criterion for single photon emission^6^.

The second-order autocorrelation function of the photons emitted by the hybrid system under consideration is given by Eq. (12). We assume that the QD is initially in its ground state, $\mathrm{|3}\rangle$, and the x-polarized driving field couples the transition $\mathrm{|2}\rangle \leftrightarrow \mathrm{|3}\rangle$ (along the *x* axis). Since the transitions $\mathrm{|2}\rangle \leftrightarrow \mathrm{|1}\rangle$ (along the *z* axis) and $\mathrm{|2}\rangle \leftrightarrow \mathrm{|3}\rangle$ (along the *x* axis) are in near-resonance with the *x*- and *z*-plasmon modes of the MNP, the surface plasmons are excited through the energy of the transitions $\mathrm{|2}\rangle \leftrightarrow \mathrm{|1}\rangle$ and $\mathrm{|2}\rangle \leftrightarrow \mathrm{|3}\rangle$. In our numerical calculations, we use the experimental values for the pure dephasing rates *γ*_1_ and *γ*_2_ reported in ref.^20^ for InAs QDs at room temperature, i.e., *γ*_1_ = *γ*_2_ = 10 *μ*eV. The spin transition rate is estimated to be *γ*_*s*_ = 1 *μeV*^48^, however, our numerical results show that the spin transition in the system under consideration has a negligible effect on the photon statistics. In the following, we explore the controllability of the photon-number statistics through the Rabi frequency of the driving field (Ω), the QD-MNP separation distance (*h*), and detuning frequency of the quantum dot transitions with respect to the surface plasmon modes (*δ*).

**Figure 5:**
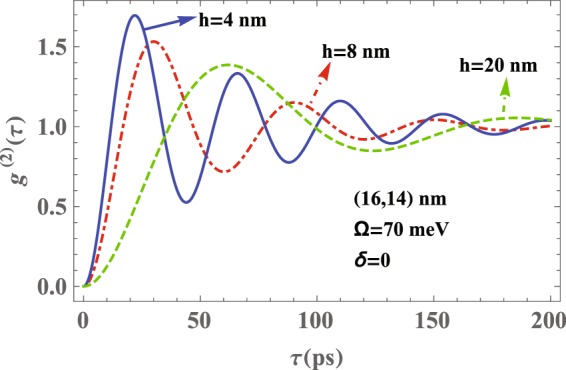
Normalized second-order autocorrelation function, *g*^(2)^(*τ*), versus the delay time *τ* for the photons emitted from a QD located in the vicinity of a (16, 14) nm nanoshell for different values of *h*. Other parameters are the same as those in Fig. 4.

To investigate the effect of the QD-MNP separation distance *h* on the normalized second-order autocorrelation function of the emitted photons, in Fig. 5 we have plotted *g*^(2)^(*τ*) against the delay time *τ* for different values of the QD-MNP separation distance. We assume that the emitter is on resonance with the LSP mode (i.e., ${\omega }_{1-}^{z(x)}$). This figure demonstrates that by increasing the QD-MNP separation distance the magnitude and the number of oscillations of *g*^(2)^(*τ*) decrease. This result can be interpreted by the LDOS of the nanoshell. The reduction of the LDOS at frequency ${\omega }_{1-}^{z(x)}$, (see Fig. 2(a) and (b)), decreases the coupling strength between the QD and plasmon modes. Consequently, the backaction of the reservoir on the QD and its re-excitation decreases as well. Moreover, by increasing the QD-MNP separation distance the antibunching time increases, because of the enhanced coupling between the QD and the surface plasmon modes compared to the coupling between the QD and the unwated mode. The oscillation periods for *h* = 4,8,20 nm are about 19.5, 22, and 78 *ps*, respectively.

**Figure 6:**
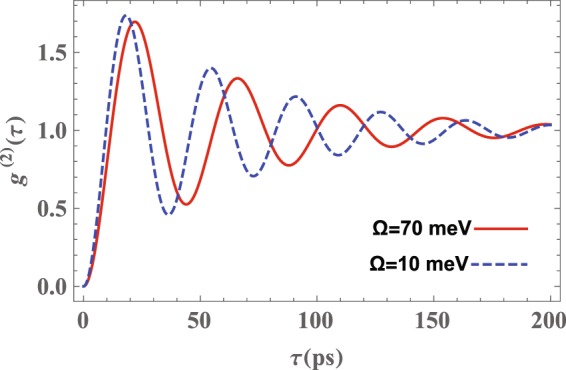
Normalized second-order autocorrelation function, *g*^(2)^(*τ*), versus the delay time *τ* for the photons emitted by a QD located at *h* = 4 nm away from a (16, 14)nm nanoshell with *ε*_*c*_ = *ε*_*b*_ = 10.95, and Δ = *ω*_23_−*ω*_*L*_ = 0 for two values of the Rabi frequency of the driving laser. Other parameters are the same as those in Fig. 4.

In Fig. 6 we have plotted the autocorrelation function *g*^(2)^(*τ*) versus the delay time *τ* for the different values of the Rabi frequency when the QD is placed at distance *h* = 4 nm from a (16, 14) nm nanoshell. Furthermore, we have assumed that the driving laser is resonantly coupled to the QD transition $\mathrm{|2}\rangle \leftrightarrow \mathrm{|3}\rangle$, i.e., Δ = 0, and the QD transitions $\mathrm{|2}\rangle \leftrightarrow \mathrm{|1}\rangle$ and $\mathrm{|2}\rangle \leftrightarrow \mathrm{|3}\rangle$ are near resonance with the LSP mode of the nanoshell, i.e., *ω*_1−_ = 0.8026 eV; more precisely, ${\omega }_{21}={\omega }_{1-}^{z}+2meV$ and ${\omega }_{23}={\omega }_{1-}^{x}+1meV$^51^. As can be seen, *g*^(2)^(*τ* = 0) = 0 and ${g}^{\mathrm{(2)}}(\tau ) > {g}^{\mathrm{(2)}}(0)$ as $\tau  > 0$. This demonstrates the presence of photon antibunching, which is definitely of quantum origin. Moreover, *g*^(2)^(*τ*) shows an oscillatory dependence on *τ*. The magnitude of these oscillations decreases as *τ* is increased and finally *g*^(2)^(*τ*) is stabilized at an asymptotic value, *g*^(2)^(*τ*) = 1. The oscillatory behavior comes from the non-Markovianity of the system evolution. In addition, the figure shows that increasing the Rabi frequency decreases the coupling strength between the QD and surface plasmon modes and, consequently, the antibunching time increases.

**Figure 7:**
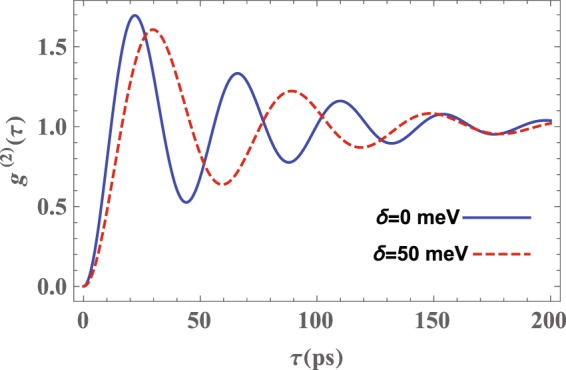
The normalized second-order autocorrelation function, *g*^(2)^(*τ*), versus the delay time *τ* for the photons emitted by a QD located at *h* = 4 nm away from a (16, 14)nm nanoshell. Other parameters are the same as those in Fig. 4.

In the next step, we investigate the impact of detuning frequency between the QD transitions $\mathrm{|2}\rangle \leftrightarrow \mathrm{|1}\rangle$ and $\mathrm{|2}\rangle \leftrightarrow \mathrm{|3}\rangle$ and two perpendicular surface plasmon modes (${\omega }_{1-}^{z(x)}$), i.e., *δ*_*z*(*x*)_, on the photon-number statistics. In Fig. 7 we have plotted *g*^(2)^(*τ*) versus the delay time *τ* for two values of the detuning frequency *δ*_*z*(*x*)_ when the QD is placed at the distance *h* = 4 nm from a (16, 14) nm nanoshell. One can see from Fig. 7 that with increasing *δ*_*z*(*x*)_ from zero (solid blue curve) to 0.05 eV (dashed red curve) the antibunching time increases.

The LDOS of a (16, 14) nm nanoshell (Fig. 2(a) and (b)) shows that the FWHM of the lowest resonance plasmon mode in energy (${\omega }_{1-}^{z(x)}$), which is coupled to the QD transition $\mathrm{|2}\rangle \leftrightarrow \mathrm{|1(3)}\rangle$, is about 0.25 eV. By taking some frequency away the summit, the intensity of LDOS is significantly weakened. Consequently, the QD interacts with the unwanted modes and this process leads to the QD dissipation and decreases the coupling strength between QD and surface plasmon modes which makes the Rabi oscillations slower.

Based on the results obtained above we can claim that the optimal situation to achieve photon antibunching in a QD-nanoshell hybrid system is one in which *h* = 4 nm, the detuning frequency between the QD transition $\mathrm{|2}\rangle \leftrightarrow \mathrm{|1(3)}\rangle$ and the surface plasmon mode (${\omega }_{1-}^{z(x)}$) is zero *δ* = 0, and the Rabi frequency is set to its minimum value. In general, Figs 5, 6 and 7 show that by decreasing the QD-MNP separation distance and the detuning *δ* along with increasing the Rabi frequency Ω one can achieve optimal photon antibunching in the system under consideration.

## Conclusions

In conclusion, we have presented a theoretical study of the photon-number statistics of a hybrid system composed of an emitter (QD) in the proximity of an MNP with non-Markovian dynamics. Our approach is based on the classical Green’s function technique and a time-convolution master equation. The InAs quantum dot is taken as a three-level Λ-configuration system in which the transition channels are orthogonal. One of the transition channels is along the *x* axis and the other is along the *z* axis. The *x* channel is coupled to a *x*-polarized classical driving field while both channels are coupled to the elementary excitations of the MNP. The statistical properties of the emitted photons are determined through the normalized second-order correlation function which can be controlled by the geometrical as well as the physical parameters of the system, including the QD-MNP separation distance, the QD-surface plasmon modes detuning, and the Rabi frequency of the *x*-polarized driving field.

We have numerically examined the memory effects on the spontaneous decay of the QD and the dynamics of the system under study. We have shown that the LDOS around the nanoshell is affected dramatically by the QD-MNP separation distance as well as the materials of the core and the embedding medium. Since the MNP affects on *x*- and *z*-polarized modes differently the anisotropic Purcell effect happens. The FWHM of the LDOS, which originates from the dissipation in the system under study as well as the intensity of the LDOS, have significant effect on the second-order correlation function. Following this viewpoint, the smaller the FWHM is, the more oscillatory behavior in the photon-number statistics is. Higher intensity of LDOS leads to more oscillatory behavior of the autocorrelation function. We have found that by increasing enough the QD-MNP separation distance and/or the QD-surface plasmon mode detuning the behavior of the system enters the Markovian regime. We have also shown that in both Markovian and non-Markovian regimes the emitted photons of the system exhibit the antibunching feature. Moreover, the QD-MNP hybrid system under study behaves as an ideal single-photon source (*g*^(2)^(*τ*) = 0), irrespective of the QD-MNP separation distance. To sum up, the results reveal that the presented hybrid system has the potential to be a highly controllable single-photon source.

## Methods

### System Hamiltonian

The total Hamiltonian of the whole system, in the rotating-wave approximation, can be written as

2 $$\begin{array}{rcl}H & = & {\sum }_{i=1}^{3}{\omega }_{i}{\sigma }_{ii}+{\sum }_{i=x,z}\int {d}^{3}{r}{\int }_{0}^{\infty }{\omega }_{i}\,{{f}}^{\dagger }({r},{\omega }_{i}).{f}({r},{\omega }_{i})d{\omega }_{i}-[{\sigma }_{21}{\int }_{0}^{\infty }d{\omega }_{x}{{d}}_{21}\mathrm{.}{E}({{r}}_{d},{\omega }_{x})+H\mathrm{.}c\mathrm{.]}\\  &  & -[{\sigma }_{23}{\int }_{0}^{\infty }d{\omega }_{z}{d}_{23}\mathrm{.}{E}({{r}}_{d},{\omega }_{z})+H\mathrm{.}c\mathrm{.}]+{H}_{D},\end{array}$$

where the first term denotes the free Hamiltonian of the QD, the second describes the Hamiltonian of the medium-assisted quantized electromagnetic field, and the third and fourth terms refer to the QD-MNP interaction Hamiltonian. Here, *σ*_*ij*_ are the Pauli operators, *f*(*r*, *ω*) and ${{f}}^{\dagger }({r},\omega )$ denote, respectively, the bosonic annihilation and creation operators for the elementary excitations of the lossy metal nanoparticle satisfying the commutation relation $[{f}({r},\omega ),{{f}}^{\dagger }(r^{\prime} ,\omega ^{\prime} )]=\delta (\omega -\omega ^{\prime} )\delta ({r}-r^{\prime} )$^62^, and *d*_*ij*_ is the transition dipole moment between *i* and *j* levels. Moreover, **E**(**r**_*d*_,*ω*) is the electric field operator at the position of the QD and is defined in the following.

The part *H*_*D*_ in the total Hamiltonian of Eq. (2) accounts for the coupling of the QD to the external driving field and is given by ${H}_{D}={\rm{\Omega }}{\sigma }_{23}{e}^{-i{\omega }_{L}t}+H.C.$ where the effective Rabi frequency is defined as ${\rm{\Omega }}=\langle {{E}}_{pump}({{r}}_{d},{\omega }_{L})\rangle \mathrm{.}{{d}}_{23}/{\varepsilon }_{eff}$ with *ε*_*eff*_ = (2*ε*_*b*_ + *ε*_*QD*_)/3*ε*_*b*_ in which *ε*_*QD*_ is the dielectric constant of the QD. In this definition, *E*_*pump*_ contains both the direct classical monochromatic field of frequency *ω*_*L*_ and amplitude **E**_*L*_(**r**_*d*_,*ω*_*L*_), and the scattered field from the MNP which would be defined through the classical dyadic Green’s function ${G}({{r}}_{d},{r}^{\prime} ,{\omega }_{L})$ as^24^

3 $${{E}}_{pump}({{r}}_{d},{\omega }_{L})={{E}}_{L}({{r}}_{d},{\omega }_{L})+{\int }_{{V}_{MNP}}G({{r}}_{d},{r}^{\prime} ,{\omega }_{L})({\varepsilon }_{MNP}({\omega }_{L})-\mathrm{1)}{{E}}_{L}({r}^{\prime} ,{\omega }_{L}){e}^{i\varphi \text{'}}{d}^{3}{r}^{\prime} .$$

Here *φ*′ is the phase change associated with the scattered laser field. Quantization of the electromagnetic fields in the presence of an absorbing and dispersive medium via the dyadic Green’s function approach leads to an explicit expression for the electric field operator in the following form^62^

4 $${E}({r},\omega )=i{(\frac{1}{\pi {\varepsilon }_{0}})}^{\mathrm{1/2}}\int {(\frac{\omega }{c})}^{2}\sqrt{{\varepsilon }_{I}({r}^{\prime} ,\omega )}{G}({r},{r}^{\prime} ,\omega )\times {f}({r}^{\prime} ,\omega ){d}^{3}{r}^{\prime} ,$$

where *ε*_*I*_(*r*, *ω*) is the imaginary part of the frequency-dependent dielectric permittivity of absorbing medium. Also, in this equation ${G}({r},{r}^{\prime} ,\omega )$ is the dyadic Green’s function of the system describing the system response at *r* to a point source at *r*′. The Green’s function is obtained through two contributions, ${G}({r},{r}^{\prime} ,\omega )={{G}}^{0}({r},{r}^{\prime} ,\omega )+{{G}}^{s}({r},{r}^{\prime} ,\omega )$ where ${{G}}^{0}({r},{r}^{\prime} ,\omega )$ is the direct contribution from the radiation sources in free-space solution and ${{G}}^{s}({r},{r}^{\prime} ,\omega )$ is the reflection contribution coming from the interaction of the dipole with the materials. In a frame rotating at the laser frequency ${\omega }_{L}^{x}$ the total Hamiltonian of Eq. (2) reads with

5 $$\begin{array}{rcl}H^{\prime}  & = & \{({\omega }_{2}-{\omega }_{L}^{x}){\sigma }_{22}+{\omega }_{3}{\sigma }_{33}\}+({\rm{\Omega }}{\sigma }_{23}+H\mathrm{.}c\mathrm{.})\\  &  & +\sum _{i=x,z}\int {d}^{3}{r}{\int }_{0}^{\infty }{\omega }_{i}{{f}}^{\dagger }({r},{\omega }_{i}\mathrm{).}{f}({r},{\omega }_{i})d{\omega }_{i},\\  &  & -[{\sigma }_{21}{e}^{i{\omega }_{L}^{x}t}{\int }_{0}^{\infty }d{\omega }_{x}{d}_{21}\mathrm{.}{E}({{r}}_{d},{\omega }_{x})+{\sigma }_{23}{e}^{i{\omega }_{L}^{x}t}{\int }_{0}^{\infty }d{\omega }_{z}{d}_{23}\mathrm{.}{E}({{r}}_{d},{\omega }_{z})+H\mathrm{.}C\mathrm{.].}\end{array}$$

### Dynamics of the system

The time evolution of the whole system which is composed of the QD, the MNP, and the coherent laser field, in the interaction picture is determined through the Liouville equation^63,64^

6 $$d{\tilde{\rho }}_{T}(t)/dt=-\,i[\tilde{H}{^{\prime} }_{Int}(t),{\tilde{\rho }}_{T}(t)].$$

Here, the Hamiltonian and the total density matrix in the interaction picture are defined as: $\tilde{H}{^{\prime} }_{Int}(t)={e}^{iH{^{\prime} }_{0}t}H{^{\prime} }_{Int}{e}^{-iH{^{\prime} }_{0}t}$ and ${\tilde{\rho }}_{T}(t)={e}^{iH{^{\prime} }_{0}t}{\rho }_{T}(t){e}^{-iH{^{\prime} }_{0}t}$. The time evolution of the total density matrix *ρ*_*T*_(*t*) in the Schrödinger picture is given by

7 $$\frac{d{\rho }_{T}(t)}{dt}=-\,i[H{^{\prime} }_{0}(t),{\rho }_{T}(t)]+{e}^{-iH{^{\prime} }_{0}t}\frac{d{\tilde{\rho }}_{T}(t)}{dt}{e}^{iH{^{\prime} }_{0}t}.$$

We assume that the reservoir is in thermal equilibrium and the states of the system and the reservoir are initially uncorrelated, so that *ρ*_*T*_(0) = *ρ*(0)⊗*R*_0_ in which *ρ*(0) and *R*_0_ stand for the initial density matrix of the system and the reservoir, respectively. By formally integrating Eq. (6), and inserting such a formal solution for ${\tilde{\rho }}_{T}(t)$ on the right hand side of Eq. (6) and after partial tracing over the reservoir degrees of freedom and applying a second-order Born approximation, the master equation of the reduced density matrix of the QD in the interaction picture, $\tilde{\rho }(t)$, is obtained as

8 $$\frac{d}{dt}\tilde{\rho }(t)=-\,T{r}_{R}{\int }_{0}^{t}dt^{\prime} [\tilde{H}{^{\prime} }_{Int}(t),[\tilde{H}{^{\prime} }_{Int}(t^{\prime} ),\tilde{\rho }(t^{\prime} )\otimes {R}_{0}]],$$

where we have used $T{r}_{R}\{\tilde{H}{^{\prime} }_{Int}(t){R}_{0}\}=0$ because the system-reservoir interaction has no diagonal elements in the representation in which the Hamiltonian of the thermal reservoir is diagonal. By substituting Eq. (8) into Eq. (7) and partial tracing over the reservoir variables one arrives at

9 $$\begin{array}{rcl}\frac{d\rho }{dt} & = & -i[H{^{\prime} }_{0S},\rho ]-T{r}_{R}{\int }_{0}^{t}dt^{\prime} \{H{^{\prime} }_{Int}\tilde{H}{^{\prime} }_{Int}(t^{\prime} -t)\rho (t^{\prime} ){R}_{0}-H{^{\prime} }_{Int}{R}_{0}\rho (t^{\prime} )\tilde{H}{^{\prime} }_{Int}(t^{\prime} -t)\\  &  & -\tilde{H}{\text{'}}_{Int}(t^{\prime} -t)\rho (t^{\prime} ){R}_{0}H{^{\prime} }_{Int}+{R}_{0}\rho (t^{\prime} )\tilde{H}{^{\prime} }_{Int}(t^{\prime} -t)H{^{\prime} }_{Int}\}.\end{array}$$

Since the plasmonic modes are excited at optical frequencies whose energy scales are much higher than the thermal energy *k*_*B*_*T*, it is reasonable to assume that the system is at zero temperature^29,30^. Therefore, we can use the following correlation functions for the reservoir operators: $T{r}_{R}[{{f}}_{i}({r},\omega ){{f}}_{i}^{\dagger }({r}^{\prime} ,\omega ^{\prime} ){R}_{0}]=\delta ({r}-{r}^{\prime} )\delta (\omega -\omega ^{\prime} )$ and $T{r}_{R}[{{f}}_{i}^{\dagger }({r},\omega ){{f}}_{i}({r}^{\prime} ,\omega ^{\prime} ){R}_{0}]=0$. On the other hand, taking a look at Eq. (9), one can recognize that this master equation is a non-Markovian master equation in the time convolution form. After calculating the integrand on the right hand side of Eq. (9) explicitly we arrive at

10 $$\begin{array}{rcl}\frac{d\rho }{dt} & = & -i[H{^{\prime} }_{0S},\rho ]+{\int }_{0}^{t}d\tau \{{\alpha }_{z}(\,-\,\tau )[\,-\,{\sigma }_{21}{\tilde{\sigma }}_{12}(\,-\,\tau )\rho (\tau )+{\tilde{\sigma }}_{12}(\,-\,\tau )\rho (\tau ){\sigma }_{21}]\\  &  & +{\alpha }_{x}(\,-\,\tau )[\,-{\sigma }_{23}{\tilde{\sigma }}_{32}(\,-\,\tau )\rho (\tau )+{\tilde{\sigma }}_{32}(\,-\,\tau )\rho (\tau ){\sigma }_{23}]+H\mathrm{.}c\mathrm{.\}}+{L}_{pure}+{L}_{spinflip},\end{array}$$

where $t-t^{\prime} =\tau$, ${\tilde{\sigma }}_{\mathrm{12(32)}}(\,-\,\tau )={e}^{-iH{^{\prime} }_{0S}\tau }{\sigma }_{\mathrm{12(32)}}\mathrm{(0)}{e}^{iH{^{\prime} }_{0S}\tau }$. By this definition ${\tilde{\sigma }}_{12}={\sigma }_{12}\mathrm{(0)}{e}^{i({\omega }_{2}-{\omega }_{L}^{x})\tau }$, for on-resonance driving (i.e., ${\omega }_{2}-{\omega }_{3}={\omega }_{L}^{x})$, ${\tilde{\sigma }}_{32}(\,-\,\tau )={\sigma }_{32}\mathrm{(0)(1}+\,\cos (2{\rm{\Omega }}\tau ))/2+{\sigma }_{23}\mathrm{(0)(1}\,-\,\cos (2{\rm{\Omega }}\tau ))/2-i\,\sin (2{\rm{\Omega }}\tau )$ $\times ({\sigma }_{22}\,-\,{\sigma }_{33})/2$, and ${\alpha }_{x(z)}(\,-\,\tau )={\int }_{0}^{\infty }J({\omega }_{x(z)}){e}^{i({\omega }_{x(z)}-{\omega }_{L}^{x})\tau }d{\omega }_{x(z)}$ in which the spectral density *J*_*x*(*z*)_(*ω*_*x*(*z*)_) is defined as

11 $${J}_{x(z)}({\omega }_{x(z)})={(\pi {\varepsilon }_{0}{c}^{2})}^{-1}{\omega }_{x(z)}^{2}{{d}}_{\mathrm{21(23)}}\mathrm{.}{\mathbb{I}}{\rm{m}}{G}({{r}}_{d},{{r}}_{d},{\omega }_{x(z)}\mathrm{).}{{d}}_{\mathrm{12(32)}}.$$

The last two terms on the right hand side of Eq. (10) correspond to the pure dephasing and spin relaxation of lower energy levels of the QD, respectively. Here, $L={\sum }_{\mu }2{L}_{\mu }\rho {L}_{\mu }^{\dagger }-{L}_{\mu }^{\dagger }{L}_{\mu }\rho -\rho {L}_{\mu }^{\dagger }{L}_{\mu }$ in which the operators ${L}_{\mathrm{2(3)}}=\sqrt{{\gamma }_{\mathrm{2(3)}}}\mathrm{|2(3)}\rangle \langle \mathrm{2(3)|}$ indicate the pure dephasing and *γ*_2_ and *γ*_3_ are the pure dephasing rates of the QD states $\mathrm{|2}\rangle$ and $\mathrm{|3}\rangle$, respectively; while ${L}_{\mathrm{1(3)}}=\sqrt{{\gamma }_{s}}\mathrm{|1(3)}\rangle \langle \mathrm{3(1)|}$ are the operators of spin relaxation and *γ*_*s*_ indicates the spin relaxation of lower energy states^65^ and it is non-radiative decay rate^66^. Furthermore, in deriving Eq. (10) we have made use of the relation $\int \varepsilon ({r},\omega )(\frac{\omega }{c}{)}^{2}{G}({{r}}_{d},{r}^{\prime} ,\omega ){{G}}^{\ast }({r}^{\prime} ,{r}{^{\prime} }_{d},\omega ){d}^{3}{r}^{\prime} ={\mathbb{I}}{\rm{m}}{G}({{r}}_{d},{{r}}_{d},\omega )$^62^.

### Photon-number statistics

One of the main purposes of the present contribution is to explore the photon-number statistics of the light emitted from the QD-MNP hybrid system. In particular, we intend to analyze the influence of the geometry of the MNP on the statistical properties of the emitted photons. The photon-number statistics can be determined by the normalized second-order photon autocorrelation function, i.e., the conditional probability of detecting the second photon at time *t* = *τ* when the first photon has already been detected at *t* = 0^63^. For a given quantum emitter system with an available excited state and a single or multiple channel(s) of relaxation, this autocorrelation function can be written as^24,38^.

12 $${g}^{\mathrm{(2)}}(\tau )=\frac{{\rho }_{22}(\tau )}{{\rho }_{22}(\infty )},$$

where ${\rho }_{22}(\tau )=\langle \mathrm{2|}\hat{\rho }(\tau \mathrm{)|2}\rangle$ and *ρ*_22_(∞) indicates the steady-state population of the excited state. Considering the initial conditions *ρ*_33_(0) = 1, *ρ*_22_(0) = *ρ*_11_(0) = 0, and *ρ*_11_ + *ρ*_22_ + *ρ*_33_ = 1 the Laplace transform of the excited-state population is obtained as

13 $${\rho }_{22}(s)=\{2{\mathbb{R}}e(\frac{A(s)P(s)}{N(s)})+2{\rm{\Omega }}{\mathbb{I}}{\rm{m}}(\frac{P(s)}{N(s)})\}/\{s+{{\rm{\Gamma }}}_{2}(s)-2{\mathbb{R}}e(\frac{A(s)M(s)}{N(s)})-2{\rm{\Omega }}{\mathbb{I}}{\rm{m}}(\frac{A(s)M(s)}{N(s)})\}\mathrm{.}$$

For convenience, we define the total spontaneous emission rate of the state $\mathrm{|2}\rangle$ as Γ_2_(*s*) = Γ_21_(*s*) + Γ_23_(*s*) in which ${{\rm{\Gamma }}}_{21}(s)=2{\mathbb{R}}e\{{\beta }_{z}(s)\}$ and ${{\rm{\Gamma }}}_{23}(s)={\mathbb{R}}e\{{\alpha }_{x}(s)+({\eta }_{x}^{\mathrm{(1)}}(s)+{\eta }_{x}^{\mathrm{(2)}}(s))/2\}$ with

14 $$\begin{array}{rcl}{\beta }_{z}(s) & = & i{\int }_{0}^{\infty }\frac{{J}_{z}({\omega }_{z})}{{\omega }_{2}-{\omega }_{z}+is}d{\omega }_{z},\\ {\alpha }_{z(x)}(s) & = & i{\int }_{0}^{\infty }\frac{{J}_{z(x)}({\omega }_{z(x)})}{{\omega }_{L}^{x}-{\omega }_{z(x)}+is}d{\omega }_{z},\\ {\eta }_{x}^{\mathrm{(1)/(2)}}(s) & = & i{\int }_{0}^{\infty }\frac{{J}_{x}({\omega }_{x})}{{\omega }_{L}^{x}-{\omega }_{x}\pm 2{\rm{\Omega }}+is}d{\omega }_{x}.\end{array}$$

Moreover, the Lamb shift is given by $\delta {\omega }_{2}={\mathbb{I}}{\rm{m}}\{2{\beta }_{z}(s)+{\alpha }_{x}(s)+({\eta }_{x}^{\mathrm{(1)}}(s)+{\eta }_{x}^{\mathrm{(2)}}(s\mathrm{))/2}\}$^67^. In Eq. (13) the functions *M*(*s*), *N*(*s*), and *P*(*s*) are defined by

15a $$M(s)={({A}^{\ast }(s))}^{2}+{{\rm{\Omega }}}^{2}+\{{{\rm{\Gamma }}}_{23}(s)-2{\gamma }_{s}\}{B}^{\ast }(s)-\{s+4{\gamma }_{s}+{{\rm{\Gamma }}}_{21}(s)\}\{{({A}^{\ast }(s)+i{\rm{\Omega }})}^{2}/(s+4{\gamma }_{s})-{B}^{\ast }(s)\},$$

15b $$N(s)=\{s+{\gamma }_{2}+{\gamma }_{3}+{\gamma }_{s}+{{\rm{\Gamma }}}_{2}(s)-i\delta {\omega }_{2}(s)\}(i{\rm{\Omega }}+{A}^{\ast }(s))+{B}^{\ast }(s)(A(s)-i{\rm{\Omega }}),$$

15c $$P(s)=\{{({A}^{\ast }(s)+i{\rm{\Omega }})}^{2}+2{\gamma }_{s}{B}^{\ast }(s)\}/\{s+4{\gamma }_{s}\},$$

where $$A(s)=({\eta }_{x}^{\mathrm{(2)}}(s)-{\eta }_{x}^{\mathrm{(1)}}(s\mathrm{))/4}$$ and $$B(s)={\alpha }_{x}(s)/2-({\eta }_{x}^{\mathrm{(2)}}(s)+{\eta }_{x}^{\mathrm{(1)}}(s))/4$$.
